# Decoding the Prognosis of Sepsis-Induced Acute Lung Injury: Machine Learning Model Development and SHAP Interpretability Analysis Based on Critical Care Databases

**DOI:** 10.3390/diagnostics16213519

**Published:** 2026-10-27

**Authors:** Yan Wang, Ke Ma, Huali Zhang, Huan Chen

**Affiliations:** 1 Sepsis Translational Medicine Key Laboratory of Hunan Province, Department of Pathophysiology, Xiangya School of Basic Medicine Science, Central South University, Changsha 410078, China; wangyanjht@126.com; 2 Chengdu Techman Software Co., Ltd., Chengdu 610041, China

**Keywords:** machine learning, sepsis-induced acute lung injury, prediction model, MIMIC-IV database, eICU-CRD database

## Abstract

**Background**: Sepsis-induced acute lung injury (SI-ALI) has high morbidity and mortality. Early prognosis prediction is critical, but traditional scoring systems lack dynamic, interpretable, and personalized capabilities. This study aimed to develop and validate an interpretable machine learning model for predicting mortality in SI-ALI. **Methods**: Data from the MIMIC-IV database (training/internal validation) and from the eICU-CRD database (external validation) were analyzed. The Lasso regression model with cross-validation was utilized for the identification of the key features. Subsequently, a series of predicted models were formulated utilizing six distinct machine learning algorithms, and the performance of the models was evaluated using various analytical tools. SHAP values were employed to interpret the machine learning models. **Results**: Among 7250 patients, eight key indicators were identified. The GBDT model achieved an internal AUC of 0.801 (95% CI: 0.775–0.827), accuracy 0.730, sensitivity 0.692, and specificity 0.739. External validation showed an AUC of 0.681 (95% CI: 0.650–0.708), accuracy 0.601, sensitivity 0.686, and specificity 0.574. However, precision, F1, and MCC were modest, indicating limited predictive accuracy. SHAP identified urine output, respiratory rate, BUN, PLR, lactate, INR, age, and anion gap as the most influential variables. **Conclusions**: The GBDT model showed modest discriminative ability and limited generalizability. It should be considered an exploratory tool requiring further prospective multicenter validation before clinical application.

## 1. Introduction

Sepsis-induced acute lung injury (SI-ALI), particularly in its most severe form—acute respiratory distress syndrome (ARDS)—remains a persistent and formidable challenge in critical care medicine. ARDS is a life-threatening syndrome characterized by the acute onset of non-cardiac pulmonary edema, severe hypoxemia, and diminished pulmonary compliance. Globally, the annual incidence of ARDS is estimated at 1.5 to 75 cases per 100,000 population, with mortality rates reaching 46.1% [1,2]. Concurrently, sepsis, defined as a life-threatening multiorgan dysfunction caused by dysregulated host responses to infection, imposes a similarly substantial disease burden. According to Global Burden of Disease studies, the age-standardized incidence rate of sepsis in 2017 was 508.4 per 100,000 population, with at least 20% of global deaths attributable to sepsis [3]. The two conditions are closely interrelated, with sepsis being one of the most common causes of ARDS; approximately 31.4% to 33.8% of sepsis patients progress to ARDS [4,5]. When sepsis and ARDS coexist, they significantly exacerbate systemic inflammatory responses and organ dysfunction, leading to a dramatic deterioration in patient prognosis and a marked increase in mortality [6]. Despite the advances in critical care support that have been witnessed in recent years, there has been minimal improvement in the mortality rates for SI-ALI patients, which remained persistently high at approximately 40% [1,7]. Consequently, the accurate identification of high-risk patients early in the course of disease is of paramount importance for guiding targeted interventions, optimizing clinical decision-making and resource allocation, and ultimately improving patient outcomes.

The advancement of medical informatics, in conjunction with the emergence of substantial public critical care databases such as the Medical Information Market for Critical Care (MIMIC) and the eICU Collaborative Research Database (eICU-CRD), signifies significant opportunities for clinical research utilizing real-world big data [8,9,10]. Concurrently, machine learning methods demonstrate immense potential in medical prognosis prediction. In comparison with conventional traditional statistical models, machine learning algorithms, including Decision Tree (DT), Gradient Boosting Decision Tree (GBDT), and Support Vector Machines (SVM), have been shown to possess the capability to expeditiously process high-dimensional, non-linear clinical data and automatically discern intricate interactions between variables [11,12,13]. Xu et al. developed a nomogram incorporating 13 clinical features (AUC: 0.811) to predict the risk of ARDS in patients with sepsis, using the MIMIC-IV database [14]. Another study utilized the MIMIC database to develop various machine learning models, which successfully predicted in-hospital mortality in patients with sepsis-associated ARDS; the random forest model performed best and identified APACHE III, bicarbonate, anion gap and systolic blood pressure as key risk factors [15]. In addition, systematic reviews have consolidated the evidence base for ML applications in sepsis, highlighting both their promise and current limitations [16].

Furthermore, advances in explainable artificial intelligence, particularly the application of the SHapley Additive exPlanations (SHAP) framework, provide powerful tools for unravelling the ‘black box’ nature of machine learning [17,18]. The SHAP methods build upon the concept of Shapley value, a concept derived from game theory, to quantify the contribution of each feature to an individual prediction outcome. This quantification is presented through intuitive visualizations, including feature importance rankings, swarm plots, and dependency graphs [19]. Jiang et al. employed SHAP for real-time, clustered risk factor analysis of sepsis and septic death in critical care, demonstrating the potential of interpretable ML for effective clinical decision-making [20]. This enhancement of transparency in models not only facilitates understanding among clinical researchers but also enables the comprehension of the directional effects (positive or negative) of key prognostic drivers, thereby establishing a linkage between data-driven discoveries and pathophysiological mechanisms, thus generating actionable clinical insights.

Despite these advances, existing studies on ML applications for predicting outcomes in sepsis-associated ARDS still have several gaps. First, many studies rely on single-center data or outdated database versions, limiting generalizability [21]. Second, external validation—particularly across geographically and institutionally diverse populations—remains insufficient, with most models validated only on internal splits of the same database [20]. Third, few studies have leveraged the updated 2023 ARDS global definition to construct prognostic models using multi-database external validation [18]. These gaps underscore the need for a more robust, interpretable, and externally validated prognostic tool that can reliably stratify SI-ALI patients across different clinical settings.

In light of the aforementioned context, the present study integrates two major international open-access critical care databases—MIMIC-IV and eICU-CRD—with a view to constructing and validating a prognostic model for patients suffering from SI-ALI. This approach employs explainable machine learning methods, informed by the new global definition of ARDS [22,23]. This study aimed to contribute to the existing literature by integrating the MIMIC-IV and eICU-CRD databases to enable the external validation of a prognostic model for SI-ALI, and by adopting the 2023 global definition of ARDS to enhance clinical relevance. A two-stage feature selection approach was employed to identify a parsimonious set of seven clinical variables, and SHAP analysis was used to improve the interpretability of the model. The model was also compared with the traditional SOFA score to explore its potential incremental value. While the findings are promising, we acknowledge that further prospective validation is needed before this tool can be considered for broader clinical application.

## 2. Methods

### 2.1. Ethics Approval and Consent to Participate

The present study utilized the MIMIC-IV (v2.2) [8] and the eICU-CRD (v2.0) [9] databases, which were used for predictive model development and internal validation, and for external validation, respectively. The MIMIC-IV database contains clinical records from more than 190,000 patients and 450,000 hospital admissions at Beth Israel Deaconess Medical Center between 2008 and 2019. The database includes, but is not limited to, patient demographics, vital signs, laboratory test results, imaging findings, medication records, and patient outcomes. The eICU-CRD database is a multicenter database containing data from over 200,000 intensive care unit admissions across 208 hospitals in the United States between 2014 and 2015. It captures clinical information comparable to MIMIC-IV with the exception of imaging findings and patient outcomes.

All methods were carried out in accordance with relevant guidelines and regulations. All the experimental protocols were approved by the Institutional Review Boards (IRBs) of both Beth Israel Deaconess Medical Center and the Massachusetts Institute of Technology. Access to both databases was granted after the first author completed the Collaborative Institutional Training Initiative (CITI) program and obtained certification in human research (Yan Wang: certification No. 58084453). All data in both databases are fully de-identified prior to public release, and the requirement for individual informed consent was waived by the respective IRBs because the study was retrospective and did not involve any direct intervention or interaction with human subjects. All methods were performed in accordance with the relevant guidelines and regulations, including the Health Insurance Portability and Accountability Act (HIPAA) and the ethical principles set forth in the Declaration of Helsinki.

### 2.2. Patient Cohort

The present study utilized MIMIC-IV and eICU-CRD databases to identify patients with SI-ALI through a sequential, time-anchored screening process for suspected infection, sepsis, and ARDS, as defined below. A suspected infection in these two databases was defined as the presence of at least one microbiological culture order within 72 h before or 24 h after the start of antibiotic therapy. The earliest time point at which these criteria were met was recorded as the time of suspected infection, irrespective of whether the culture result was positive. Patients meeting these criteria were flagged as suspected infection. The diagnostic criteria for sepsis are in accordance with the Third International Consensus on Sepsis and Septic Shock (Sepsis-3). Sepsis-3 defines sepsis as a Sequential Organ Failure Assessment (SOFA) score of ≥2, in conjunction with clinical indications of infection or suspected infection [23]. In the absence of pre-admission SOFA baseline data, the first SOFA score recorded within the first 24 h of ICU admission was used, implicitly assuming that the baseline SOFA score was 0. To ensure temporal association between infection and organ dysfunction, the SOFA end time had to fall within a time window extending from 48 h before to 24 h after the suspected infection time. Then, the diagnostic criteria for ARDS are in accordance with the new global definition, which encompasses universal criteria applicable to all types of ARDS, as well as criteria applicable to specific types of ARDS [22]. In this context, ARDS was identified based on oxygenation metrics and respiratory support parameters recorded within the first day of ICU admission, with stratification according to ventilation status. Non-intubated ARDS was defined as a PaO_2_/FiO_2_ ratio of ≤300 mmHg or a SpO_2_/FiO_2_ ratio of ≤315 (if SpO_2_ was ≤97%) under the following conditions: an oxygen flow rate of ≥30 L/min when using a high-flow nasal cannula, or a positive end-expiratory pressure of ≥5 cmH_2_O when using non-invasive ventilation or continuous positive airway pressure. Invasively ventilated patients were required to meet at least one of the oxygenation impairment criteria listed above without any additional respiratory support. The definition of SI-ALI was as follows: it was both the presence of sepsis and ARDS on the first day of admission to the ICU. Finally, the primary outcome of this study was in-hospital mortality, which was defined as death occurring at any time during the initial hospitalization. This outcome was chosen as it is the most consistently documented and least ambiguous endpoint in both databases.

**Figure 1:**
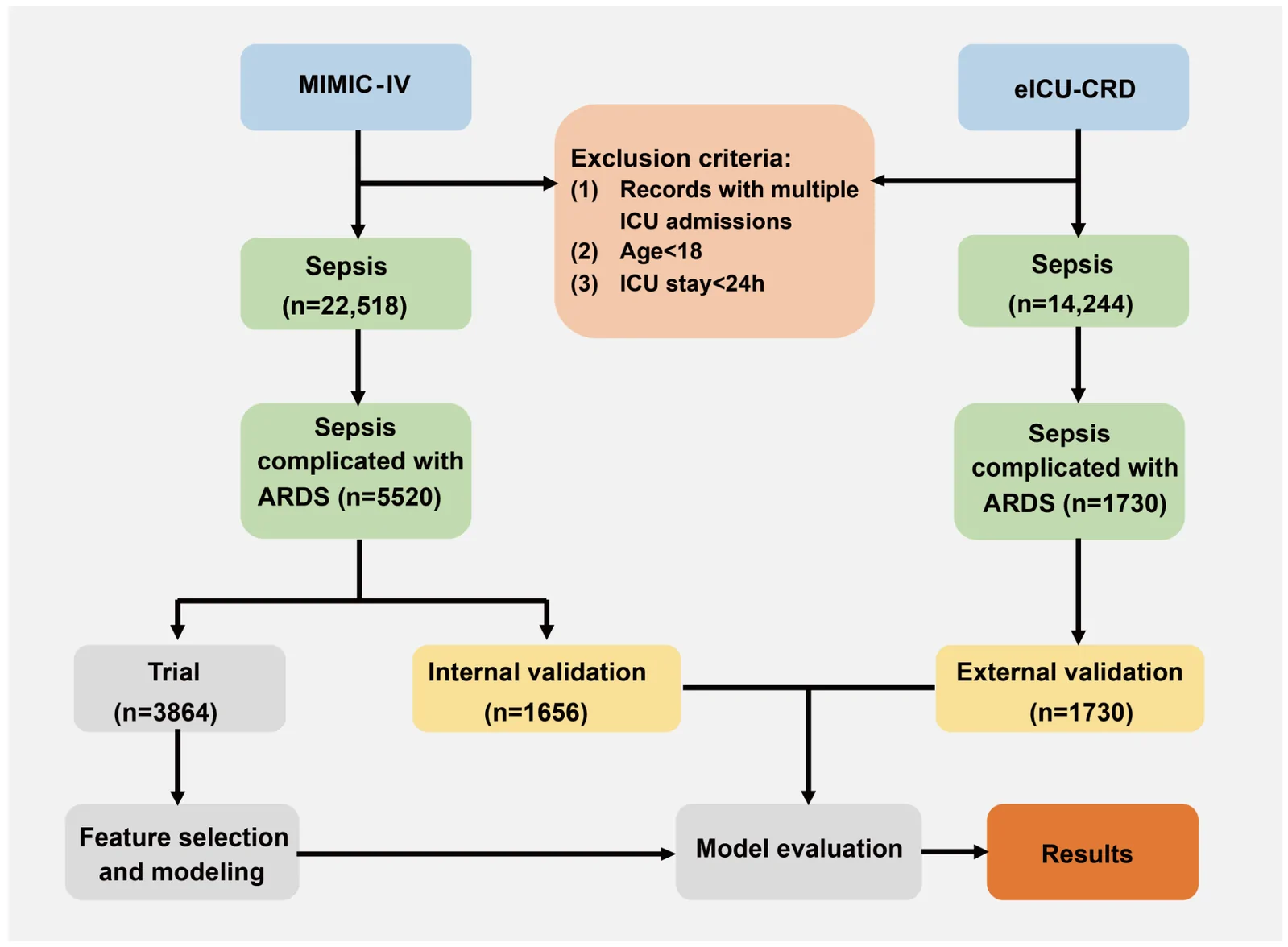
Workflow diagram.

In order to ensure data quality, to mitigate the influence of very short-term ICU admissions, and to maintain a homogeneous adult study population, the following exclusion criteria were applied: (1) Records with multiple ICU admissions (with the focus being on the first ICU stay for each patient). (2) ICU stay of less than 24 h. (3) Age of less than 18 years. The flowchart illustrating the patient cohort inclusion is presented in Figure 1.

### 2.3. Data Extraction

The Structured Query Language was utilized in Navicat Premium software (v 17.0) to extract the clinical data of patients with sepsis complicated by ARDS within 24 h of admission to the ICU. For the MIMIC-IV database, which served as the development and internal validation cohort, a comprehensive set of variables was initially extracted across the following categories: 1. The demographic information collected included age, gender, and body mass index (BMI). 2. The patient’s medical history includes the following comorbidities: diabetes, congestive heart failure, malignant cancer, and chronic pulmonary disease. 3. The laboratory test results are listed below: hemoglobin, hematocrit, white blood cell count, platelet count, platelet-to-lymphocyte ratio (PLR), neutrophil-to-lymphocyte ratio (NLR), lymphocyte-to-monocyte ratio, sodium, glucose, bicarbonate, blood urea nitrogen (BUN), potassium, calcium, anion gap, bilirubin, creatinine, albumin, C-reactive protein, international normalized ratio (INR), lactate, pH, oxygen saturation, PaO_2_/FiO_2_, SpO_2_/FiO_2_. 4. Vital signs: respiratory rate (RR), heart rate, systolic blood pressure (SBP), mean blood pressure, diastolic blood pressure, temperature, urine output. 5. Disease scores: SOFA, Acute Physiology Score III (APSIII), Glasgow Coma Scale. 6. Advanced life support therapy details: continue renal replacement therapy, invasive mechanical ventilation, vasoactive drugs. In instances where continuous variables were measured on multiple occasions, the mean value was employed for the purposes of analysis. Conversely, binary variables were analyzed based on their documented values.

Variables from the two databases were harmonized according to the following principles: (1) units: all laboratory values were converted to consistent units (e.g., BUN: mg/dL, creatinine: mg/dL, lactate: mmol/L); (2) timing: all variables were extracted as the mean value within the first 24 h of ICU admission; (3) ventilation: invasive mechanical ventilation was defined based on documented ventilation status; (4) urine output: total urine output (mL) was extracted for the first 24 h; (5) outcome: in-hospital mortality was uniformly defined as death during the index hospitalization; (6) SpO_2_ vs. SaO_2_: SpO_2_ (pulse oximetry) was used as the primary oxygen saturation variable, while SaO_2_ (arterial blood gas) was used when available.

Prior to analysis, the extracted data underwent a rigorous preprocessing pipeline to ensure robustness and validity. Initially, variables with missing values exceeding 40% of the cohort were excluded from subsequent analysis to circumvent potential bias from excessive imputation. For the remaining variables, with missing rates ranging from 0% to 32%, the dataset was randomly divided into a training set and an internal validation set using stratified random sampling in a 7:3 ratio to ensure the preservation of the outcome distribution. The random seed was fixed at 123 to ensure reproducibility. Multiple imputation was then performed exclusively on the training set using the MICE package in R software (v 4.4.2), generating five imputed datasets over ten iterations via chained equations. Predictive mean matching was utilized for continuous numeric variables, and logistic regression for binary variables. To prevent data leakage, the imputation models fitted on the training set were subsequently applied to the internal validation and external validation sets to impute their missing values. The five-fold imputation structure was thus maintained across the training and test sets, and all five imputed datasets were retained for subsequent analysis. Subsequently, in order to address multicollinearity, a variance inflation factor (VIF) analysis was conducted on each imputed training set. Variables with a VIF > 10 were considered to exhibit severe multicollinearity and were thus iteratively removed from the feature set. The process yielded a final, refined set of baseline characteristics that were used for statistical analysis and modelling.

### 2.4. Statistical Analysis and Modeling

The normality of continuous variables was assessed using the Shapiro–Wilk test. Continuous variables that were not normally distributed were expressed as median and interquartile range, and subsequently compared using the Mann–Whitney U test. Binary variables were summarized as numbers and percentages and then compared using either the Chi-square test or Fisher’s exact test, as appropriate. All analyses were performed using R software (v 4.4.2), with a statistical significance level set at *p* < 0.05.

The development of the model was executed within the Python framework (v 3.7.16). In the context of the five imputed training sets, the Least Absolute Shrinkage and Selection Operator with cross-validation (Lasso-CV) was applied exclusively to the training set to eliminate redundant variables. This was followed by Recursive Feature Elimination (RFE) to iteratively remove the least important predictors and obtain a more discriminative and parsimonious subset of variables. The feature selection process was executed independently within each imputed dataset. The final feature set was determined by a frequency-based voting rule across the five imputations (a feature was retained if it was selected in at least 60% of the imputed datasets). This procedure ensured that no information from the internal validation set was utilized during feature selection. The construction of the model was achieved through the utilization of six machine learning models, namely GBDT, SVM, DT, Adaptive Boosting (AdaBoost), Logistic Regression (LR), and K-Nearest Neighbors (KNN), with the relevant hyperparameters detailed in Table S1. For each imputed dataset, every candidate model was trained on the corresponding imputed training set and used to predict the corresponding imputed test set. The final predicted probability for each patient was obtained by averaging the predicted probabilities across the five imputed datasets (prediction averaging). The predictive performance of the models was comprehensively evaluated using a range of metrics, including the Receiver Operating Characteristic (ROC) curve, Area Under the Curve (AUC), the precision–recall curve and area under the PR curve (PR-AUC, which is robust under class imbalance), accuracy, precision, sensitivity, specificity, the F1-score, and the Matthews correlation coefficient (MCC). Pairwise AUC comparisons were performed using the paired DeLong’s test to evaluate the statistically significant difference in predictive performance among models. Furthermore, calibration curves were utilized to evaluate prediction reliability, and decision curve analysis (DCA) was conducted to assess clinical utility. In order to enhance the interpretability of the models and mitigate their inherent “black-box” nature, the SHAP framework was employed to quantify and visualize the contribution of each feature to individual predictions. The model that demonstrated the most optimal performance was the ultimate prediction model for in-hospital mortality in patients with sepsis complicated by ARDS.

## 3. Results

### 3.1. Baseline Characteristics

The present study included 7250 patients with SI-ALI who met the predefined inclusion criteria. The data presented herein were obtained from two extensive clinical databases: MIMIC-IV and eICU-CRD. The MIMIC-IV database contributed 5520 patients (training and internal validation sets), comprising 4491 survivors and 1029 non-survivors, while the eICU-CRD database provided 1730 patients (external validation set), including 1307 survivors and 423 non-survivors. The baseline characteristics of patients in each subgroup, based on the first of the five multiply imputed datasets, are summarized in Tables S2 and S3. Notably, the baseline characteristics between these two cohorts revealed several differences. For instance, patients in the MIMIC-IV cohort were slightly younger and had a higher proportion of male patients. MIMIC-IV patients exhibited higher rates of diabetes and congestive heart failure, whereas the proportion of patients with chronic pulmonary disease was comparable between the two databases. Patients in the eICU-CRD database had higher median glucose levels, lower BUN levels in survivors, and lower anion gap values. The utilization of invasive mechanical ventilation was higher in MIMIC-IV, whereas the use of vasoactive drugs was more prevalent in MIMIC-IV.

A comparative analysis of baseline characteristics revealed statistically significant differences between the survival and non-survival groups for the majority of clinical variables. Patients in the non-survival group were significantly older than those in the survival group (*p* < 0.001). The laboratory test results indicated that metabolic disturbances were more severe in the deceased group. These disturbances manifested as elevated BUN, lactate levels, and anion gap, while bicarbonate concentration and pH were significantly lower. Vital sign monitoring revealed that patients in the deceased group exhibited diminished physiological stability, marked by elevated respiratory rate, tachycardia, diminished blood pressure, and markedly reduced urine output. Furthermore, the disease severity scores of patients such as SOFA and APSIII in the non-survival group were significantly higher than those in the survival group. Consequently, patients in the non-survival group exhibited a greater need for intensive life-support therapy, encompassing a higher proportion of invasive mechanical ventilation, vasopressor use, and continuous renal replacement therapy. These substantial disparities in baseline characteristics underscore the close association between the severity of early organ dysfunction following ICU admission and the risk of in-hospital mortality.

### 3.2. Selection of Key Features

To address the methodological challenges inherent in such studies, including the presence of redundant data and selection bias, a comprehensive data preprocessing procedure was implemented prior to the selection of features. Initially, the elimination of variables lacking statistical significance was conducted through univariate analysis. Subsequent multicollinearity assessment revealed that hematocrit and MBP exhibited severe multicollinearity and were therefore excluded from further analysis. It is noteworthy that established clinical severity scores (SOFA and APSIII) were intentionally excluded during the development of the model to enable direct comparison between the predictive model and the SOFA score benchmark. Following the implementation of the aforementioned preprocessing procedures, a candidate feature set comprising 34 variables was retained for the purpose of subsequent predictive modeling.

**Figure 2:**
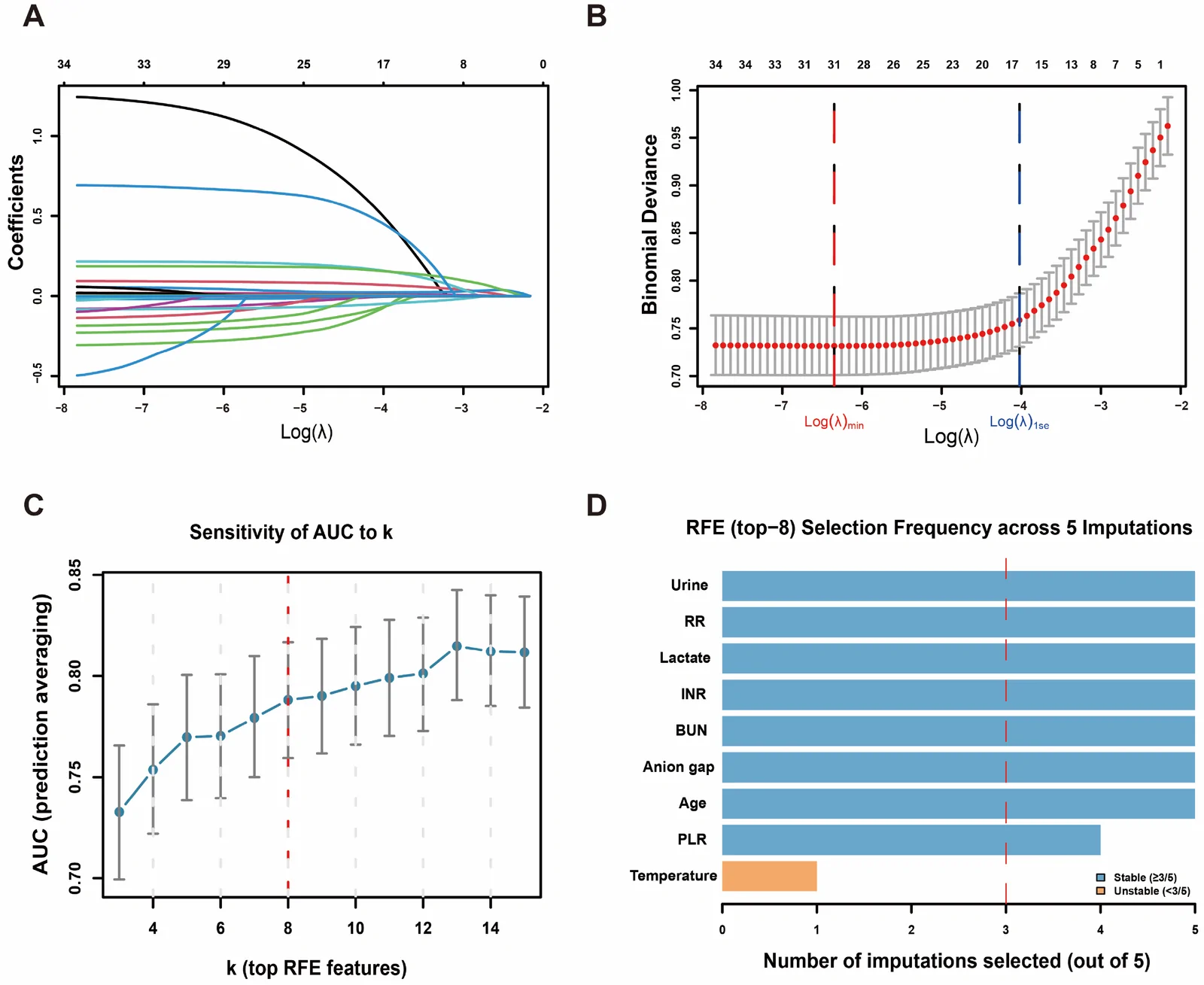
The feature selection process using Lasso-CV and RFE methods on the training sets. (**A**) Variable coefficient paths across penalty parameter values. (**B**) Selection of the optimal parameter through cross-validation error curves. Panels (**A**,**B**) were derived from the first imputed training set to illustrate the Lasso-CV process. (**C**) Sensitivity analysis of the number of features retained by RFE (*k*) on model performance, with predictions combined by averaging across five imputed training sets. (**D**) Selection frequency of the top eight features identified by RFE across five imputed training sets. Note: BUN: blood urea nitrogen; INR: international normalized ratio; PLR: platelet-to-lymphocyte ratio; RR: respiratory rate.

To develop a model that exhibits enhanced predictive performance and clinical applicability, a two-stage feature selection approach was employed. The initial stage of the process employed Lasso-CV for the purpose of initial dimensionality reduction. This was achieved by reducing the coefficients of features that were found to be weakly correlated to zero. When applied to the first imputed training set, Lasso-CV identified 17 key features from the 34 candidate variables (see Figure 2A,B). The selected variables comprised the following: age, sex, BMI, history of malignancy, hemoglobin, anion gap, INR, BUN, lactate, oxygen saturation, PaO_2_/FiO_2_, PLR, NLR, RR, body temperature, minute ventilation, and urine output. The second stage of the process involved the application of RFE to refine the variables selected by Lasso-CV. To assess the effect of the number of features retained by RFE (*k*) on model performance, a sensitivity analysis was conducted across *k* = 3 to 15. For each value of *k*, the top *k* features were selected from the full ranking of the RFE. Generalized linear models were then fitted separately on the five imputed datasets. The predictions were then averaged in order to create a composite prediction, and the AUC was calculated based on the averaged predictions. The sensitivity analysis indicated that *k* = 8 provided a reasonable balance between predictive performance and model parsimony (see Figure 2C). The final set of eight features determined by frequency voting across the five imputed datasets was as follows: urine, RR, lactate, INR, BUN, anion gap, age, and PLR (see Figure 2D).

### 3.3. Model Performance and Its Comparison with Existing Scoring Systems

**Figure 3:**
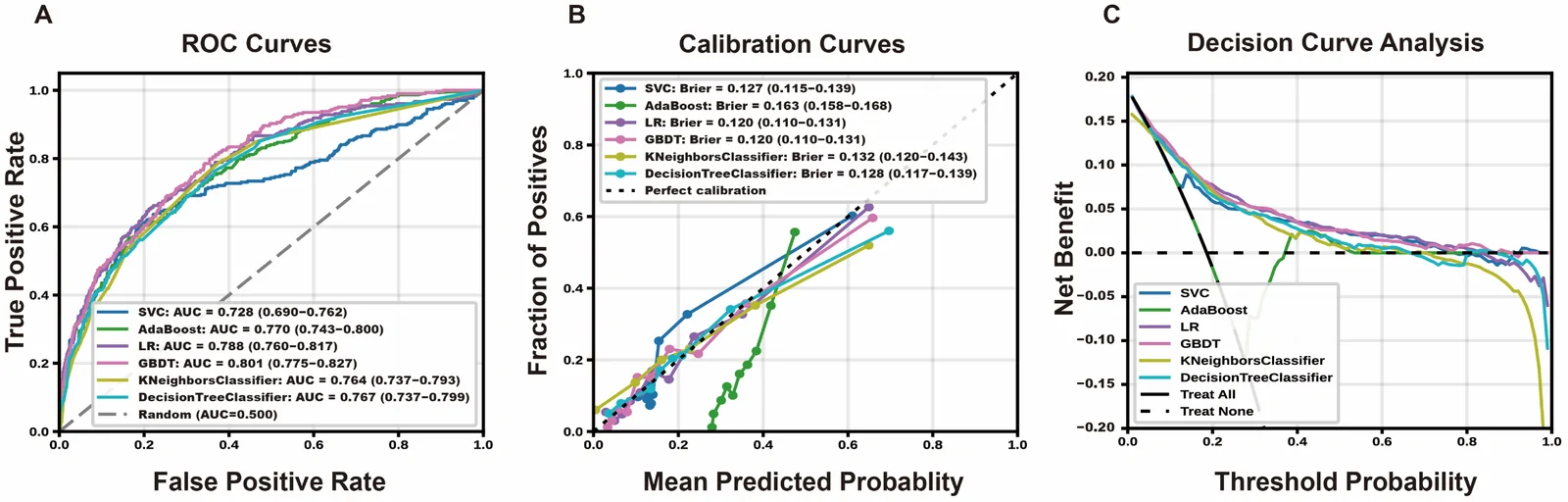
The ROC curves (**A**), calibration curves (**B**), and DCA curves (**C**) of six machine learning models on the internal validation set. Note: AdaBoost: Adaptive Boosting; DCA: decision curve analysis; DT: Decision Tree; GBDT: Gradient Boosting Decision Tree; KNN: K-Nearest Neighbors; LR: Logistic Regression; ROC: Receiver Operating Characteristic; SVM: Support Vector Machines.

The set of variables that were identified through the process of feature selection was subsequently entered into the model for the purposes of internal validation. To assess the comparative efficacy of these models, a variety of metrics were employed, including the AUC, accuracy, sensitivity, and specificity. The metrics were calculated alongside their 95% confidence intervals (CI), ensuring a comprehensive and precise evaluation of the models’ performance. The specific results are displayed in Table 1 and Table S4, Figure 3A. The findings suggest that, across all models, GBDT exhibited the most superior predictive capability, with an AUC of 0.801 (95% CI: 0.775–0.827), while concurrently maintaining competitive accuracy (0.730), sensitivity (0.692), and specificity (0.739), precision (0.378), F1-score (0.488), MCC (0.353), and PR-AUC (0.524). The LR model demonstrated comparable performance, exhibiting a second-highest degree of AUC. To ascertain whether GBDT was statistically superior to other models, we performed pairwise comparisons of AUC using DeLong’s test. The findings of the study demonstrated that the GBDT exhibited a marked superiority over the AdaBoost, KNN, SVM, and DT algorithms (*p* < 0.001; see Table S5). However, no statistically significant differences were observed between the GBDT and the LR model. The calibration curves for the model demonstrated that the probabilities predicted by the GBDT exhibited strong concordance with the observed actual mortality rates (Brier score = 0.120; Figure 3B). The DCA curve further emphasized the clinical utility of GBDT, as its net benefit consistently exceeded that of the “all-treatment” and “all-no-treatment” strategies (see Figure 3C). Consequently, GBDT was selected as the primary model for external validation.

**Figure 4:**
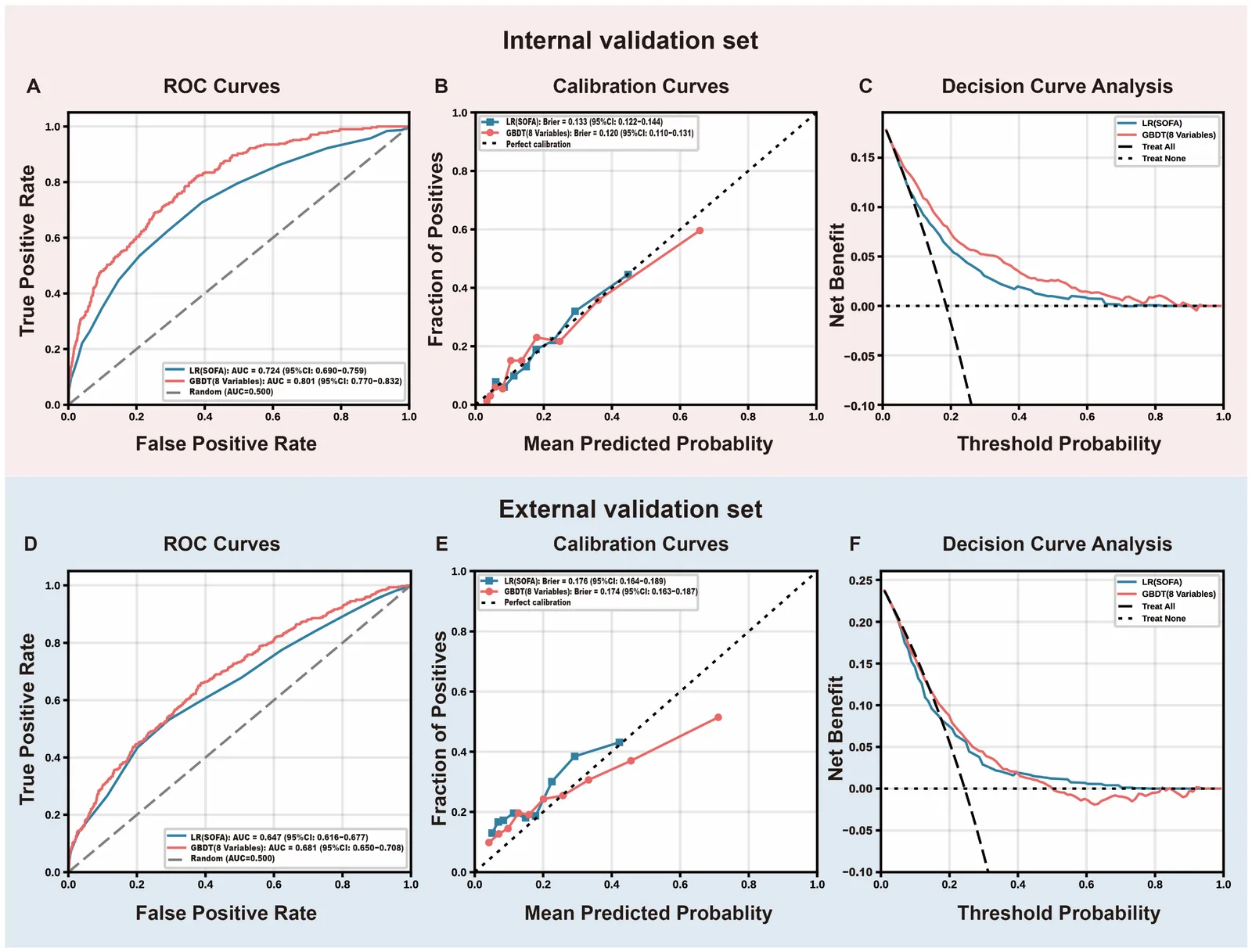
Comparison between the LR(SOFA) model and the 8-variable-based GBDT model on both internal and external validation sets. (**A**–**C**) The internal validation set comprises: ROC curves (**A**), calibration curves (**B**), and DCA curves (**C**). (**D**–**F**) The external validation set comprises: ROC curves (**D**), calibration curves (**E**), and DCA curves (**F**). Note: DCA: decision curve analysis; GBDT: Gradient Boosting Decision Tree; ROC: Receiver Operating Characteristic; LR(SOFA): SOFA logistic regression model.

Subsequently, an external evaluation of the GBDT was conducted, with the eICU-CRD database being utilized as the primary source of data. As demonstrated in Table 2 and Figure 4D, the model exhibited good generalization capability, attaining an AUC of 0.681 (95% CI: 0.650–0.708) on the external validation set, with an accuracy of 0.601, a sensitivity of 0.686, a specificity of 0.574, a precision of 0.342, a F1-score of 0.457, a MCC of 0.223, and a PR-AUC of 0.437. In comparison with the internal validation, the AUC on the external validation exhibited a decrease of approximately 12%. The calibration curve demonstrated good performance on the external validation set, as evidenced by the Brier score of 0.174 (see Figure 4E). Furthermore, the DCA curve substantiated the GBDT model’s clinical applicability on external data (see Figure 4F). The findings indicated that GBDT demonstrated good internal performance while exhibiting favorable discriminatory power and external calibration. This suggests that GBDT is a viable tool for decision-making processes aimed at predicting patient mortality risk.

To assess the incremental value of the new model, a comparison was made between its predictive performance and that of a logistic regression model based on the total SOFA score (LR(SOFA)), a traditional scoring system commonly used in ICUs. The 8-variable-based GBDT model performed well on both the internal (see Figure 4A–C) and external (see Figure 4D–F) validation sets, exhibiting higher AUC, enhanced accuracy and better clinical applicability when compared with the SOFA score. These results indicate that the 8-variable GBDT is capable of predicting the prognosis of patients with SI-ALI with relatively greater accuracy.

### 3.4. Model Explanation

**Figure 5:**
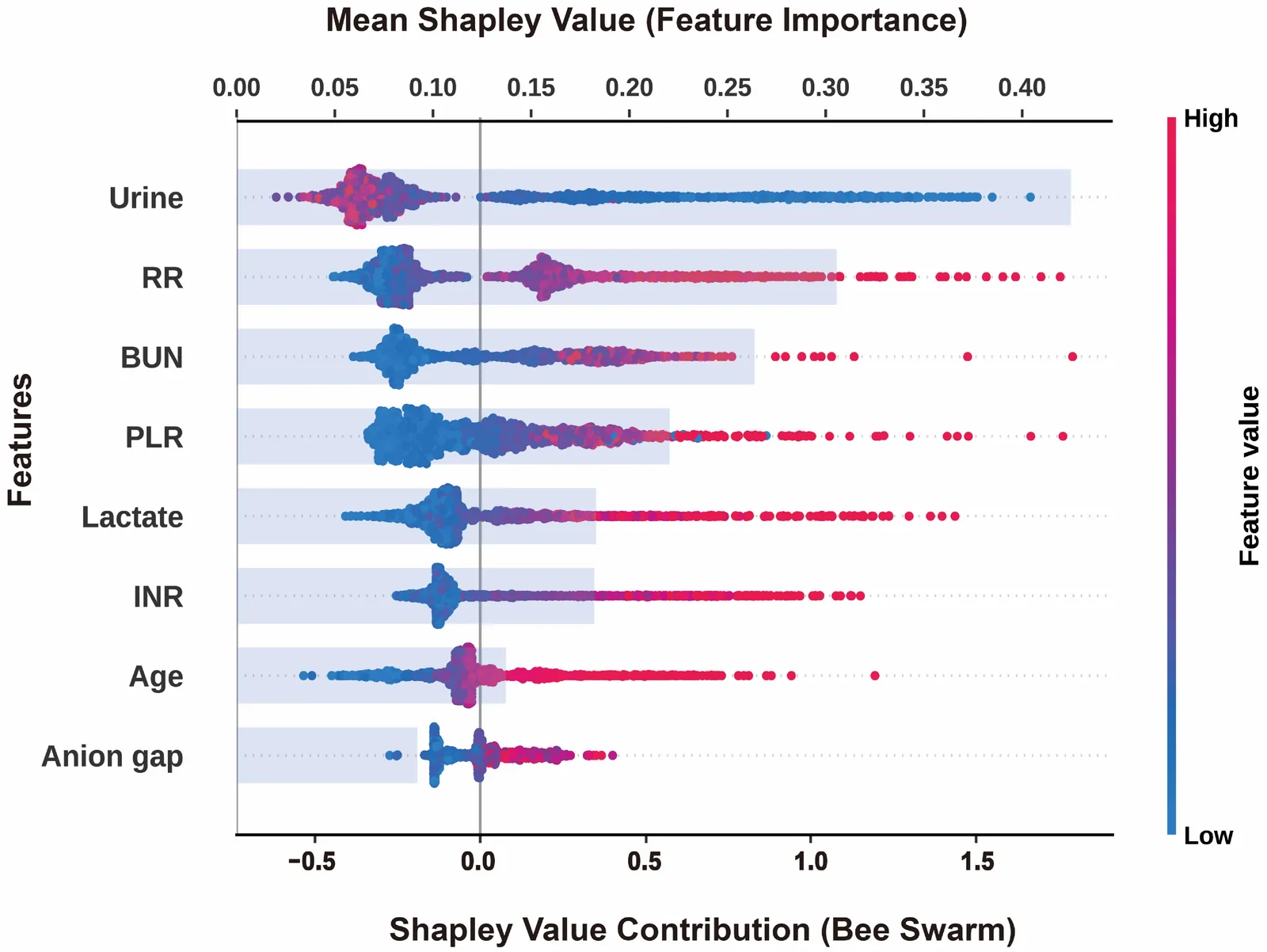
The SHAP summary plot that visualizes feature importance and contribution direction. The horizontal axis represents SHAP values, indicating the magnitude and direction of each variable’s impact on the model’s prediction, while the vertical axis lists key variables. Bar chart (light blue): the degree of feature importance. Honeycomb plot: the distribution of each feature’s SHAP values across all samples. Red dots represent high feature values, and blue dots represent low feature values. Note: RR: respiratory rate; BUN: blood urea nitrogen; PLR: platelet-to-lymphocyte ratio; INR: international normalized ratio.

To further elucidate the predictive logic of the GBDT and to identify key predictors, a SHAP analysis was conducted. As illustrated by the bar chart in Figure 5, the clinical features under consideration were collectively responsible for the prognostic decision, or feature importance. The findings suggest that urine output exerts the most significant influence on the model output, with RR ranking as the second most important factor. It is evident that a range of additional features, including but not limited to BUN, PLR, lactate, INR, age, and anion gap, also make significant contributions to the predictive results.

The honeycomb plot (SHAP value distribution plot) in Figure 5 further elucidates the direction of contribution of these key features and their nonlinear relationships. A thorough analysis of the eight features based on SHAP importance reveals that reduced urine output, increased RR, elevated BUN, elevated PLR, elevated lactate, prolonged INR, advanced age, and increased anion gap are all core risk factors for SI-ALI. Specifically, the risk of death increases significantly when urine output is below 1100 mL/24 h, RR exceeds 21 breaths per minute, BUN is above 24.33 mg/dL, PLR is above 209.7, lactate levels are above 2.5 mmol/L, INR is greater than 1.56, age exceeds 78 years, or the anion gap is above 14.67 mEq/L.

**Figure 6:**
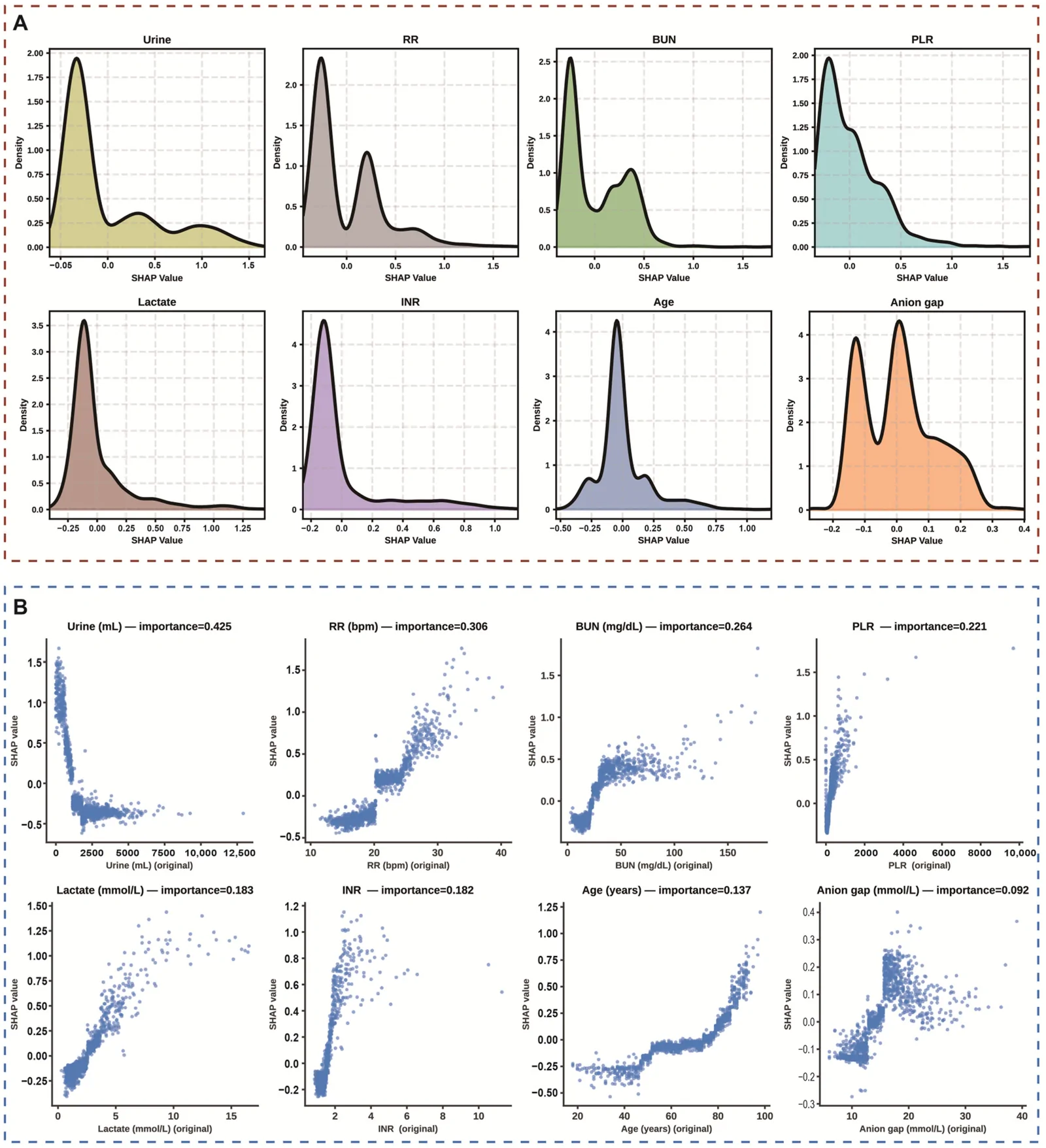
SHAP value distribution (**A**) and partial dependence plots (**B**) of the GBDT model for each feature. Note: RR: respiratory rate; BUN: blood urea nitrogen; PLR: platelet-to-lymphocyte ratio; INR: international normalized ratio.

The thresholds were determined in the following manner: initially, a SHAP dependency plot was generated for each feature, illustrating how the SHAP value (contribution to the risk of death) varies with the feature’s value. Secondly, bias dependency plots were overlaid to visualize the marginal effect of each feature on the predicted probability of death. Thirdly, the inflection points at which the SHAP value changes from negative (protective) to positive (increased risk) were identified; this represents the threshold at which the effect of the feature on the risk of death changes. For instance, when urine output is below approximately 1100 mL over a 24 h period, the SHAP value remains consistently positive; for RR, this threshold is 21 breaths per minute; the remaining features follow a similar pattern (Figure 6 and Table S6).

## 4. Discussion

In this study, a GBDT model was developed and internally validated for predicting in-hospital mortality in patients with SI-ALI using the MIMIC-IV database. The model was subsequently validated using the eICU-CRD databases. In the process of internal validation, the GBDT model attained an AUC of 0.801, thereby demonstrating superiority over the following models: LR, AdaBoost, DT, KNN and SVM. The SHAP analysis indicated eight key predictors: urine output, RR, BUN, PLR, lactate, INR, age and anion gap. It is important to note that threshold values were determined for each predictor, below or above which the risk of death increased significantly. The findings of this study provide a clinically interpretable framework for the early risk stratification of SI-ALI.

Firstly, our GBDT model achieved a moderate-to-good discriminative ability. The internal validation AUC was 0.801, with a sensitivity of 0.692 and specificity of 0.739. However, precision, F1-score, MCC, and PR-AUC values are relatively low. This discrepancy can be primarily attributed to the significant class imbalance in our dataset. Specifically, the class distribution is heavily skewed, with the majority class (survival) accounting for approximately 81.4% (*n* = 4491) of the total samples, while the minority class (death) constitutes only 18.6% (*n* = 1029), resulting in an approximate 4.4:1 ratio. In such highly imbalanced scenarios, precision, F1-score, MCC, and PR-AUC are particularly sensitive to the minority class. In contrast, AUC is threshold-independent and less affected by changes in class proportions.

Secondly, it is evident that the eight features identified by SHAP analysis align well with the established pathophysiology of SI-ALI and are readily available in routine ICU care [24,25,26]. The clinically suggestive thresholds identified through SHAP analysis—such as urine output < 1100 mL/24 h, RR > 21 breaths/min, BUN > 24.33 mg/dL, PLR > 209.7, lactate > 2.5 mmol/L, INR > 1.56—warrant further discussion. It is imperative to interpret these thresholds not as definitive clinical cutoffs, but rather as descriptive patterns. The methodology employed in the identification of these thresholds comprised the visual inspection of SHAP dependence plots in conjunction with partial dependence plots. This approach revealed inflection points at which feature contributions transitioned from protective to risk-increasing. The approach is consistent with recent studies employing SHAP for threshold identification in critical care settings [12,20]. Urine output is a direct and sensitive indicator of renal perfusion and microcirculatory status. In accordance with the findings of preceding studies [27], the results of this study confirm that oliguria is not merely a marker of acute kidney injury but also a reflection of systemic hemodynamic instability and microcirculatory dysfunction, making it a strong predictor of poor outcomes. With regard to respiratory function, tachypnea constitutes an early compensatory response to hypoxemia and respiratory distress in ARDS. The threshold of RR > 21 breaths/min is approaching the upper limit of normal and may serve as an early warning sign before the onset of overt respiratory failure. This finding is consistent with the established correlation between elevated RR and unfavorable outcomes in sepsis and ARDS [28,29]. Elevated BUN often indicates reduced glomerular filtration (prerenal or renal) or heightened catabolism, both of which are associated with sepsis severity and increased mortality [30,31]. PLR integrates thrombotic and inflammatory responses: elevated PLR indicates platelet activation and lymphocyte depletion, both of which are features of sepsis-induced immunothrombosis and immune suppression [32]. Lactate is a well-established marker of tissue hypoxia and anaerobic metabolism, and its prognostic value in sepsis and ARDS has been validated in multiple studies [33,34]. In the context of ARDS, local pulmonary inflammation can further elevate lactate levels, compounding systemic acidosis and organ failure [35]. Furthermore, prolonged INR indicates coagulation dysfunction, which may precede overt disseminated intravascular coagulation. In sepsis, activation of coagulation and suppression of fibrinolysis contribute to microthrombosis and organ failure [36]. Our findings support monitoring INR as part of early risk assessment. While these pathophysiological interpretations are plausible and consistent with the existing literature, it is important to emphasize that SHAP analysis, by itself, does not establish causal relationships. Rather, it provides a hypothesis-generating framework for identifying which features the model relies on to formulate its predictions. These findings should therefore serve as a springboard for future mechanistic studies and prospective validations to confirm the clinical utility of these biomarkers.

Thirdly, in comparison with the LR(SOFA) model, our 8-variable-based GBDT model achieved better performance on the databases used in this study, likely due to its ability to capture nonlinear relationships and feature interactions effectively. However, the moderate 12% decrease in AUC from internal (0.801) to external (0.681) warrants careful consideration. Such performance degradation is commonly observed when models trained on one population are transported to another, and it may be attributable to several sources of heterogeneity between MIMIC-IV and eICU-CRD. Firstly, a substantial discrepancy was observed between the two databases in terms of patient demographics and clinical characteristics. The MIMIC-IV cohort exhibited a younger demographic, a higher proportion of male patients, and a higher prevalence of diabetes and congestive heart failure. Conversely, the eICU-CRD cohort demonstrated higher median glucose levels, lower anion gap values, and a higher in-hospital mortality rate (24.4% vs. 18.6%). Secondly, disparities in data collection methodologies, variable definitions, and measurement protocols across data sources may result in alterations to the information content of features that appear to be identical. For instance, variations in the availability and recording of oxygenation variables (SaO_2_ vs. SpO_2_), minute ventilation, and urine output have the potential to affect the performance of models. Thirdly, treatment practices differed substantially between cohorts. Mechanical ventilation and the administration of vasoactive drugs were more prevalent in MIMIC-IV, reflecting potential differences in institutional practice patterns and patient acuity. Notwithstanding, an AUC of 0.681 remains within an acceptable range, thus indicating that the core pathophysiological associations captured by the model demonstrate limited stability across different populations. Nevertheless, the findings indicate that the direct extrapolation of the model to new centers exhibiting significant healthcare system or regional variations may lead to overfitting. Consequently, recalibration or fine-tuning using local data is imperative prior to clinical implementation.

This study has several strengths. The use of two large databases (MIMIC-IV and eICU-CRD) for model development and external validation enhances the generalizability of our findings and mitigates the potential biases associated with single-center studies. The integration of SHAP analysis adds a layer of interpretability that is often lacking in conventional machine learning models, making the predictions more actionable for clinicians. Our findings are also consistent with prior studies, which have identified lactate, respiratory rate, and acid-base parameters as important prognostic markers in sepsis and ARDS [4,5,18,27,37,38]. The present study extends these findings by quantifying the relative importance and directional effects of these features using SHAP, and by highlighting the clinical relevance of readily available variables such as urine output, BUN, PLR, and INR.

Nevertheless, this study is subject to the following limitations. Firstly, the inherent limitations of a retrospective study design must be acknowledged. Despite the meticulous selection of high-quality public databases, it is imperative to acknowledge the inherent limitations concerning the avoidance of selection bias and issues pertaining to missing or inconsistent data records. Secondly, our study was confined to adult patients (aged ≥18 years), and excluding pediatric populations limits the generalizability of our findings to younger age groups. Additionally, patients with ICU stays of less than 24 h were excluded, which may restrict the applicability of our model to individuals experiencing rapid clinical deterioration or early death. Furthermore, the current model relies on static data collected within the first 24 h of ICU admission, whereas the clinical trajectory of SI-ALI is dynamic and can evolve substantially over time. Therefore, before our model can be more widely adopted in clinical practice, it is essential to validate its performance in broader, more diverse populations and to develop dynamic prediction models that incorporate longitudinal time-series data. Thirdly, the model’s external validation AUC of 0.681, while acceptable, suggests that there is room for improvement in predictive performance. The moderate external performance may reflect differences in patient demographics, clinical practices, and data collection methodologies across the two databases. It may also indicate that the 8-variable model, while parsimonious, may not fully capture all the nuanced factors that contribute to mortality in SI-ALI. Further research, including larger-scale prospective validation studies and the exploration of more advanced ensemble learning techniques, will be necessary to enhance model robustness and generalizability. Finally, and most importantly, while our study demonstrates the potential of the GBDT model to predict mortality in SI-ALI, we did not evaluate whether the use of this model to guide clinical decisions actually leads to improved patient outcomes. Prospective interventional studies, ideally using a randomized controlled trial design, are urgently needed to determine whether model-guided risk stratification and early intervention can translate into tangible clinical benefits, such as reduced mortality, shorter ICU stays, or improved quality of life.

## 5. Conclusions

In summary, the present study developed and internally validated an interpretable machine learning model for predicting in-hospital mortality in patients with SI-ALI using the MIMIC-IV database, with external validation in the eICU-CRD database. The GBDT model demonstrated good discriminative ability in internal validation, but exhibited a decline in performance during external validation. Despite the identification of eight routinely available predictors and the provision of insights into their directional effects by SHAP analysis, the relatively low precision, F1 score, and MCC indicate that the model’s predictive accuracy—especially its ability to correctly identify true positive cases—is limited. The metrics indicate the presence of a certain proportion of false positives, as well as relatively weak agreement between predicted and observed outcomes.

Consequently, the model should be regarded as an exploratory, proof-of-concept instrument at this stage, rather than a reliable clinical decision aid. The findings indicate the difficulties inherent in the application of machine learning models to heterogeneous ICU populations and data sources. In order to confirm the model’s predictive accuracy, generalizability, and clinical utility, prospective multicenter validation studies with standardized data collection and model recalibration are required. It is recommended that future research explore dynamic, time-series data and assess whether model-guided interventions can meaningfully improve patient outcomes. Only after such rigorous validation can this model be considered for integration into clinical decision-support workflows.

## Figures and Tables

**Table 1:** Predictive performance of different machine learning models on the MIMIC-IV database.

	AUC	Accuracy	Sensitivity	Specificity	Precision	F1-Score	MCC	PR_AUC
GBDT	0.801 (0.775–0.827)	0.730 (0.711–0.752)	0.692 (0.641–0.744)	0.739 (0.716–0.763)	0.378 (0.338–0.419)	0.488 (0.449–0.529)	0.353 (0.307–0.404)	0.524 (0.466–0.580)
LR	0.788 (0.760–0.817)	0.692 (0.670–0.714)	0.724 (0.675–0.775)	0.685 (0.660–0.710)	0.344 (0.309–0.379)	0.467 (0.428–0.505)	0.326 (0.281–0.371)	0.514 (0.460–0.575)
AdaBoost	0.771 (0.743–0.800)	0.668 (0.645–0.692)	0.731 (0.684–0.780)	0.654 (0.628–0.679)	0.326 (0.290–0.360)	0.451 (0.412–0.489)	0.304 (0.258–0.350)	0.462 (0.409–0.523)
DT	0.767 (0.737–0.799)	0.716 (0.695–0.741)	0.643 (0.591–0.697)	0.733 (0.709–0.760)	0.355 (0.317–0.395)	0.457 (0.417–0.497)	0.309 (0.261–0.358)	0.465 (0.408–0.526)
KNN	0.764 (0.737–0.793)	0.688 (0.668–0.711)	0.727 (0.675–0.775)	0.679 (0.656–0.705)	0.342 (0.306–0.377)	0.465 (0.426–0.503)	0.324 (0.279–0.368)	0.428 (0.379–0.488)
SVM	0.728 (0.690–0.762)	0.725 (0.705–0.746)	0.653 (0.598–0.704)	0.741 (0.719–0.766)	0.366 (0.328–0.406)	0.469 (0.426–0.508)	0.325 (0.276–0.373)	0.491 (0.436–0.549)

**Table 2:** Predictive performance of the GBDT model on the eICU-CRD database.

Model	AUC	Accuracy	Sensitivity	Specificity	Precision	F1-Score	MCC	PR-AUC
GBDT	0.681 (0.650–0.708)	0.601 (0.577–0.625)	0.686 (0.638–0.727)	0.574 (0.546–0.600)	0.342 (0.311–0.374)	0.457 (0.421–0.490)	0.223 (0.175–0.267)	0.437 (0.389–0.489)

## Data Availability

This study was performed with the data from the MIMIC-IV (v2.2) and the eICU-CRD (v2.0). Even though datasets are de-identified, restrictions have been imposed on data sharing since they contain sensitive information. Conventions are signed for researchers before any access to the data. The first author of the present study, Yan Wang, has become a certified user of https://physionet.org/, completed the necessary training, and sign the data usage agreement for this project (Certificate No.: 58084453). The first author can provide details regarding data access procedures upon reasonable request.

## Supplementary Materials

The following supporting information can be downloaded at: https://www.mdpi.com/article/10.3390/diagnostics16213519/s1, Table S1: Hyperparameter configurations for each machine learning model; Table S2: Base characteristics of sepsis-induced acute lung injury patients from the MIMIC-IV database; Table S3: Baseline characteristics of sepsis-induced acute lung injury patients from the eICU-CRD database; Table S4: The confusion matrices of the GBDT model; Table S5: Pairwise comparisons of AUC between GBDT and other machine learning models using DeLong’s test; Table S6: Cut-off values of eight features in the partial dependence plots.

## Abbreviations

The following abbreviations are used in this manuscript:

AdaBoost	Adaptive Boosting
ARDS	acute respiratory distress syndrome
AUC	Area Under the Curve
BUN	blood urea nitrogen
BMI	body mass index
CI	confidence interval
CITI	Collaborative Institutional Training Initiative
DCA	decision curve analysis
DT	Decision Tree
eICU-CRD	eICU Collaborative Research Database
GBDT	Gradient Boosting Decision Tree
HIPAA	Health Insurance Portability and Accountability Act
INR	international normalized ratio
KNN	K-Nearest Neighbors
IRBs	Institutional Review Boards
Lasso CV	Least Absolute Shrinkage and Selection Operator with Cross-Validation
LR	Logistic Regression
MIMIC	Medical Information Market for Critical Care
NLR	neutrophil-to-lymphocyte ratio
PLR	platelet-to-lymphocyte ratio
ROC	Receiver Operating Characteristic
RR	respiratory rate
SaO_2_	arterial oxygen saturation
SBP	systolic blood pressure
SHAP	SHapley Additive exPlanations
SI-ALI	sepsis-induced acute lung injury
SOFA	Sequential Organ Failure Assessment
SVM	Support Vector Machines
