# Supplementary material for: Decoding the Prognosis of Sepsis-Induced Acute Lung Injury: Machine Learning Model Development and SHAP Interpretability Analysis Based on Critical Care Databases
Source: Diagnostics (Basel). 2026 Oct 27;16(21):3519. doi: 10.3390/diagnostics16213519 (PMC13761567; doi:10.3390/diagnostics16213519)
Supplement: Supplementary file 1 [file diagnostics-16-03519-s001.zip › diagnostics-4543488-supplementary.pdf]

Table S1. Hyperparameter configurations for each machine learning model.

**Model**	**Hyperparameters**
SVM	C=1
LR	C=1
KNN	n_neighbors=5
GBDT	n_estimators=100, learning_rate=0.05, max_depth=3, min_samples_split=2, min_samples_leaf =1, subsample=1
DT	max_depth=5, min_samples_split=2, min_samples_leaf=1
AdaBoost	n_estimators=50, learning_rate=0.05

Table S2. Base characteristics of sepsis-induced acute lung injury patients from the MIMIC-IV database.

Variables	Survival (*n* =4,491)	Non-survival (*n* =1,029)	*P*-value
**Demographics**			
Age, years	66.00(56.00-76.00)	71.00(59.00-81.00)	<0.001
Gender, n (%)			<0.001
Male	2834 (63.1)	559 (54.3)	
Female	1654 (26.9)	470 (45.7)	
Body mass index (BMI), kg/m^2	30.59 (26.64-35.43)	29.03 (24.71-34.21)	<0.001
**Comorbidities**			
Diabetes, n (%)			<0.05
Yes	1480 (33.0)	296 (28.8)	
No	3011 (67.0)	733 (61.2)	
Congestive heart failure, n (%)			<0.05
Yes	1364 (30.4)	350 (34.0)	
No	3127 (59.6)	679 (66.0)	
Malignant cancer, n (%)			<0.001
Yes	428 (9.5)	196 (19.0)	
No	4063 (90.5)	833 (81.0)	
Chronic pulmonary disease, n (%)			0.39
Yes	1410 (31.4)	338 (32.8)	
No	3081 (68.6)	691 (67.2)	
**Laboratory test**			
Hemoglobin (Hb), g/dL	10.75 (9.55-12.20)	10.30 (8.92-11.93)	<0.001
Hematocrit, K/uL	32.42 (28.82-36.87)	31.91 (27.51-37.10)	<0.01
White blood cell (WBC), K/uL	12.62 (9.40-16.37)	13.57 (9.33-18.65)	<0.001
Platelet (PLT), K/uL	181.33 (135.42-240.50)	177.50 (108.67-248.50)	<0.01
Platelet-to-Lymphocyte ratio (PLR)	141.15 (81.96-250.17)	196.94 (107.56-353.66)	<0.001
Neutrophil-to-Lymphocyte ratio (NLR)	7.11 (4.30-12.50)	11.31 (6.31-20.67)	<0.001
Lymphocyte-to-Monocyte ratio (LMR)	2.35 (1.33-4.52)	1.61 (0.94-3.11)	<0.001
Sodium, mEq/L	139.00 (136.67-141.00)	138.67 (135.50-142.50)	0.91
Glucose, mg/dL	100.00 (85.00-121.00)	107.00 (85.00-136.00)	<0.001
Creatinine, mg/dL	1.00 (0.75-1.42)	1.47 (0.95-2.26)	<0.001
Bicarbonate (HCO3), mEq/L	23.20 (21.00-25.50)	21.00 (17.67-24.50)	<0.001
Blood urea nitrogen (BUN), mg/dL	19.25 (14.00-29.23)	31.25 (20.00-48.33)	<0.001
Potassium, mEq/L	4.23 (3.93-4.60)	4.30 (3.90-4.80)	<0.001
Calcium, mg/dL	8.20 (7.80-8.63)	8.12 (7.56-8.60)	<0.001
Anion gap, mEq/L	13.50 (11.50-16.00)	16.20 (13.75-19.50)	<0.001
International normalized ratio (INR)	1.25 (1.15-1.40)	1.43 (1.20-1.98)	<0.001
Lactate, mmol/L	1.80 (1.30-2.46)	2.45 (1.57-4.08)	<0.001
pH	7.37 (7.32-7.40)	7.33 (7.27-7.39)	<0.001
oxygen saturation (SaO2), %	97.50 (95.83-99.00)	96.00 (94.14-98.00)	<0.001
PaO2/FiO2, mmHg	176.25 (134.67-217.81)	139.00 (101.05-186.33)	<0.001
SpO2/FiO2, mmHg	172.61 (147.62-199.50)	164.12 (123.91-198.00)	<0.001
**Vital signs**			
Respiratory rate (RR), times/min	19.00 (17.00-22.00)	22.00 (19.00-25.00)	<0.001
Heart rate (HR), times/min	85.00 (77.00-96.00)	91.00 (78.00-104.00)	<0.001

Systolic blood pressure (SBP), mmHg	113.03 (106.33-121.45)	108.48 (101.32-117.85)	<0.001
Mean blood pressure (MBP), mmHg	75.28 (70.66-81.17)	72.96 (67.83-79.04)	<0.001
Diastolic blood pressure (DBP), mmHg	59.94 (54.86-66.04)	58.65 (52.56-65.41)	<0.001
Temperature (T), ℃	36.96 (36.68-37.32)	36.86 (36.51-37.27)	<0.001
Urine output, mL	1700 (1101-2430)	917 (423-1632)	<0.001
**Disease scores**			
SOFA	6.00 (5.00-9.00)	10.00 (7.00-13.00)	<0.001
APSIII	44.00 (32.00-59.00)	68.00 (52.00-89.00)	<0.001
GCS	15.00 (14.00-15.00)	15.00 (13.00-15.00)	0.15
**Advanced life support therapy**			
Continue renal replacement therapy, n (%)			<0.001
Yes	61 (1.4)	72 (7.0)	
No	4430 (98.6)	957 (93.0)	
Invasive mechanical ventilation, n (%)			<0.001
Yes	3733 (83.1)	971 (94.4)	
No	758 (16.9)	58 (5.6)	
Vasoactive drugs, n (%)			<0.001
Yes	2578 (57.4)	708 (68.8)	
No	1913 (42.6)	321(31.2)	

Table S3. Baseline characteristics of sepsis-induced acute lung injury patients from the eICU-CRD database.

Variables	Survival (n =1307)	Non-survival (n =423)	*P*-value
**Demographics**			
Age, years	65.00 (55.00-76.00)	70.00 (59.00-78.00)	<0.001
Gender, n (%)			0.87
Male	721 (55.2)	231 (54.6)	
Female	586 (44.8)	192 (45.4)	
Body mass index (BMI), kg/m^2	27.92 (23.74-34.17)	27.86 (23.25-33.08)	0.35
**Comorbidities**			
Diabetes, n (%)			0.75
Yes	182 (13.9)	62 (14.7)	
No	1125 (86.1)	361 (85.3)	
Congestive heart failure, n (%)			0.74
Yes	732 (56.0)	242 (57.2)	
No	575 (44.0)	181 (42.8)	
Malignant cancer, n (%)			<0.01
Yes	30 (2.3)	21 (5.0)	
No	1277 (97.7)	402 (95.0)	
Chronic pulmonary disease, n (%)			0.81
Yes	266 (20.4)	89 (21.0)	
No	1041 (79.6)	334 (79.0)	
**Laboratory test**			
Hemoglobin (Hb), g/dL	10.83 (9.35-12.50)	10.15 (8.79-12.00)	<0.001
Hematocrit, %	33.00 (28.62-38.40)	31.10 (26.98-36.77)	<0.001
White blood cell (WBC), K/uL	12.75 (9.05-17.80)	13.70 (8.85-20.33)	0.07
Platelet (PLT), K/uL	199.00 (140.14-272.00)	185.50 (115.30-266.84)	<0.05
Platelet-to-Lymphocyte ratio (PLR)	203.16 (121.30-342.74)	203.74 (108.48-348.20)	0.7
Neutrophil-to-Lymphocyte ratio (NLR)	10.30 (5.71-18.59)	11.20 (6.13-19.42)	0.08
Lymphocyte-to-Monocyte ratio (LMR)	1.55 (0.91-2.80)	1.47 (0.86-2.86)	0.50
Sodium, mEq/L	139.00 (136.00-142.00)	138.67 (135.00-142.85)	0.86
Glucose, mg/dL	139.33 (112.84-173.00)	146.50 (114.00-185.92)	<0.05
Creatinine, mg/dL	1.15 (0.78-1.94)	1.65 (1.00-2.55)	<0.001
Bicarbonate (HCO3), mEq/L	24.00 (20.67-27.00)	21.75 (18.12-26.00)	<0.001
Blood urea nitrogen (BUN), mg/dL	24.75 (15.50-41.00)	33.00 (21.00-50.50)	<0.001
Potassium, mEq/L	4.10 (3.73-4.57)	4.15 (3.80-4.70)	0.08
Calcium, mg/dL	8.17 (7.65-8.68)	8.00 (7.47-8.60)	<0.01

Anion gap, mEq/L	11.00 (8.00-15.00)	13.50 (9.75-17.50)	<0.001
International normalized ratio (INR)	1.23 (1.10-1.45)	1.40 (1.17-1.85)	<0.001
Lactate, mmol/L	1.60 (1.15-2.50)	2.17 (1.37-4.20)	<0.001
pH	7.35 (7.29-7.41)	7.33 (7.25-7.41)	<0.001
oxygen saturation (SaO2), %	96.10 (93.15-98.24)	95.12 (91.92-98.00)	<0.05
PaO2/FiO2, mmHg	206.06 (143.69-285.00)	170.00 (110.67-258.48)	<0.001
SpO2/FiO2, mmHg	172.00 (129.56-221.41)	139.29 (103.48-193.47)	<0.001
**Vital signs**			
Respiratory rate (RR), times/min	21.00 (17.00-24.00)	21.00 (19.00-25.00)	<0.001
Heart rate (HR), times/min	91.00 (79.00-103.00)	94.00 (81.00-108.00)	<0.01
Systolic blood pressure (SBP), mmHg	113.10 (103.57-125.72)	107.88 (99.85-120.02)	<0.001
Mean blood pressure (MBP), mmHg	77.00 (69.35-85.54)	72.63 (66.28-79.98)	<0.001
Diastolic blood pressure (DBP), mmHg	62.83 (56.09-70.34)	59.67 (53.27-65.54)	<0.001
Temperature (T), ℃	36.96 (36.62-37.40)	36.88 (36.44-37.30)	<0.001
Urine output, mL	1484(836-2325)	1025(418-1915)	<0.001
**Disease scores**			
SOFA	7.00 (4.00-9.00)	9.00 (6.00-12.00)	<0.001
APSIII	64.00 (47.00-85.00)	84.00 (59.50-108.00)	<0.001
GCS	12.00 (9.00-15.00)	11.00 (7.00-15.00)	<0.001
**Advanced life support therapy**			
Continue renal replacement therapy, n (%)			<0.001
Yes	30 (2.3)	32 (7.6)	
No	1277 (97.7)	391 (92.4)	
Invasive mechanical ventilation, n (%)			<0.001
Yes	769 (58.8)	308 (72.8)	
No	538 (41.2)	115 (27.2)	
Vasoactive drugs, n (%)			<0.001
Yes	343 (26.2)	187 (44.2)	
No	964 (73.8)	236 (55.8)	

Table S4. The confusion matrices of the GBDT model.

MIMIC-IV		Actually	
Predicted		Negative	Positive
	Negative	995	352
	Positive	95	213

Table S5. Pairwise comparisons of AUC between GBDT and other machine learning models using DeLong's test.

Comparison	AUC (GBDT)	AUC (Comparator)	ΔAUC	Z statistic	P-value
GBDT vs SVC	0.8012	0.7278	+0.0733	5.720	<0.001
GBDT vs AdaBoost	0.8012	0.7705	+0.0307	4.634	<0.001
GBDT vs LR	0.8012	0.7882	+0.0129	1.691	0.091
GBDT vs KNN	0.8012	0.7642	+0.0370	3.302	0.001
GBDT vs DT	0.8012	0.7661	+0.0350	4.525	<0.001

Table S6. Cut-off values of eight features in the partial dependence plots.

Feature	Cut-off value
Urine output, mL	1100
Respiratory rate (RR), times/min	21.00
Blood urea nitrogen (BUN), mg/dL	24.33
Platelet-to-Lymphocyte ratio (PLR)	209.70
Lactate, mmol/L	2.50
International normalized ratio (INR)	1.56
Age, years	78.00
Anion gap, mEq/L	14.67
